# Fiber Nanoarchitectonics for Pre-Treatments in Facile Detection of Short-Chain Fatty Acids in Waste Water and Faecal Samples

**DOI:** 10.3390/polym13223906

**Published:** 2021-11-11

**Authors:** Guozhe Deng, Li Xie, Shengjia Xu, Xuejun Kang, Jizheng Ma

**Affiliations:** 1 Key Laboratory of Child Development and Learning Science of Ministry of Education of China, School of Biological Science and Medical Engineering, Southeast University, Nanjing 210096, China; dengguozhe0608@163.com; 2 College of Animal Science and Food Engineering, Jinling Institute of Technology, Nanjing 210038, China; 11206104@163.com; 3 The Research Center of Military Exercise Science, Army Engineering University of PLA, Nanjing 211101, China; xushengjia510@126.com

**Keywords:** adsorbent, conducting polymer, electrospinning, polyacrylonitrile, solid phase extraction

## Abstract

Short-chain fatty acids (SCFAs) are among the active metabolites in biological process both in the intestinal tract and the bioconversion of organic wastes, which has resulted in various human diseases and environmental problems. In order to accurately detect SCFAs, we introduced a novel extraction sorbent. Electrospun polyacrylonitrile (PAN) nanofiber membrane was synthesized, then poly (3, 4-Ethylenedioxythiophene) (PEDOT) was deposited onto the surface of electrospun PAN nanofibers by in situ polymerization. The morphology of the composite PAN/PEDOT nanofiber was characterized by scanning electronic microscopy (SEM) and FTIR spectrum. PAN/PEDOT was used to isolate and concentrate the SCFAs in waste water and fecal samples before gas chromatography mass spectrometry (GC-MS) analysis. The analytical method was evaluated systematically, and low limits of detection (LODs) of 0.34–0.87 μg/L and good linearity (R^2^ ≥ 0.9953) were obtained. The method was applied successfully for the determination of SCFAs in waste water and fecal samples, with good recovery (87.5–104.6%) and satisfactory reproducibility (relative standard deviation: 6.5–14.1%). The results indicated that the proposed method can be used as a potential approach for the determination of SCFAs with high sensitivity in waste water and biological samples.

## 1. Introduction

Short-chain fatty acids (SCFAs) are monocarboxylic aliphatic acids with 2 to 8 carbon atoms. As important active metabolites in biological processes, the analysis of SCFAs is significant in studies of health and disease in the intestinal tract [1]. Additionally, in the production of anaerobic biodegradation of organic compounds, SCFAs are important intermediates in the conversion of organic waste to methane [2]. SCFAs served as a carbon source for denitrifying bacteria [3] and phosphorus-accumulating organisms (PAOs) [4] can be used in wastewater treatment for the biological removal of nitrogen and phosphorus. Domestic sewage contains various organic compounds that are also potential sources for resource recovery [5]. Bioconversion of these wastes into value-added products to minimize the negative environmental impact will benefit the development of society. SCFAs originating from anaerobic fermentation of organic wastes could solve the problem of carbon source shortages in waste water treatment plants (WWTPs) to improve the operating performances of biological nutrient removal [6]. Therefore, the accurate determination of SCFAs’ concentration at different stages of wastewater treatment and in the environment is important.

Generally, sample preparation is a crucial step for analytical chemistry, especially in the case of a complex specimen such as environmental or biological samples [7,8]. Solid phase extraction (SPE) is often used as the sample preparation method. SCFAs usually followed with complex biological matrixes, and their strong polarity and volatility make them difficult to extract. Liquid–liquid extraction and derivatization were usually used in the preparation of SCFAs [9], and filtration as a physical pretreatment was also exploited to reduce the presence of contaminants from the complex matrix [10]. However, liquid–liquid extraction may cause loss of analytes, and derivatization is time-consuming and may introduce new derivatives into the analysis, which requires the use of toxic organic solvents and reagents for further purification [11]. Solid phase microextraction (SPME) is also an option for the extractions of SCFAs [12], but SPME fiber is fragile and expensive, and sometimes requires longer time to extract trace analytes compared with classical solvent extraction.

Electrospinning is a versatile technique to produce nanofibers [13], and various polymer solutions can be used to fabricate nanofibers with diameters from nano-scale to micro-scale [14]. Electrospun nanofiber has three-dimensional morphology, a large surface area-to-volume ratio, and certain toughness [15] and has been studied widely in bone tissue engineering [16], CO_2_ capture [17], and immunoassay [18], among others. Besides, nanofibers have great potential as sorbents in solid-phase extraction [19] and thin film microextraction [20].

As an environmentally stable conducting polymer, poly(3,4-ethylenedioxythiophene) (PEDOT) has high conductivity and electrical stability [21] and is widely used in energy storage [22,23], catalysis [24], electronics [25], and functional coatings [26,27]. PEDOT was also applied in solid phase extraction [28,29], but, to the best of our knowledge, few reports concerned the extraction of SCFAs with PEDOT.

In this work, a new pretreatment method based polyacrylonitrile-poly(3,4-Ethylenedioxythiophene) (PAN/PDEOT) electrospun nanofiber was developed to isolate and concentrate SCFAs from waste water. PAN/PDEOT nanofiber can be easily fabricated in aqueous solution and used as the extraction phase of SPE. Aliphatic acids (acetic acid, propionic acid, isobutyric acid, butyric acid, isovaleric acid, valeric acid, hexanoic acid, and heptanoic acid) were selected as model compounds in the research. The method validation was investigated systematically, and this method was coupled to gas chromatography–mass spectrometry (GC–MS) to determine eight aliphatic acids in waste water.

## 2. Materials and Methods

### 2.1. Chemicals

Polyacrylonitrile (PAN, average MW: 150,000), polystyrene (PS, average MW: 192.000), 3, 4-Ethylenedioxythiophene (EDOT), N, N-Dimethylformamide (DMF), iron(III) chloride (FeCl_3_, ≥99.9%), ammonium persulfate (APS, ≥99.99%), pyrrole (99%), acetic acid (AA, ≥99.8%), propionic acid (PA, ≥99.5%), isobutyric acid(IBA, ≥99.5%), butyric acid (BA, >99.5%), isovaleric acid (IVA, ≥99.5%), valeric acid (VA, ≥99.5%), hexanoic acid (HXA, ≥99.5%), and heptanoic acid (HPA, 98%) were purchased from Shanghai Aladdin Biochemical Technology Co., Ltd. (Shanghai, China). 2-Ethylbutyric acid used as an internal standard and ethanol (GC grade) were obtained from Sigma-Aldrich (St. Louis, MO, USA). Hydrochloric acid (37%, analytical grade) was purchased from Sinopharm Chemical Reagent Co., Ltd. (Shanghai, China). All ultrapure water (18.2 MΩ·cm) used was obtained from a Millipore Milli-Q Plus system.

### 2.2. Apparatus

Thermo Trace 1300-ISQ GC-MS system was used to analyze the SCFAs, while a high-voltage power supply (DW-P403-1AC, Tianjin, China) and a syringe pump were used for the electrospinning. A field emission scanning electron microscope (FESEM, Zeiss Ultra Plus) was utilized to investigate the morphology and structure of the prepared nanofiber mats. Fourier transform infrared (FTIR) spectra were recorded using a Thermo Nicolet iS5 spectrometer (Madison, WI, USA) in the range of 400–4000 cm^−1^.

### 2.3. Fabrication of PAN/PEDOT and PS/PPY Nanofiber

**Figure 1:**
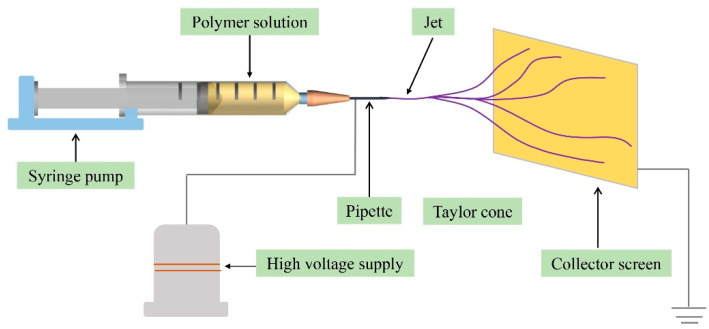
Schematic diagram of the electrospinning device.

A certain amount of PS or PAN was added into N, N-Dimethylformamide (DMF) under vigorous magnetic stirring until it was completely dissolved, forming a 10% (*w*/*v*) solution. Then, 5 mL of the prepared polymer solution was electrospun using a home-made electrospinning machine (Figure 1) at the feeding rate of 1 mL/h under an applied voltage of 20 kV. The collect screen was 10 cm from the feeding needle. A constant temperature (23 ± 2 °C) and relative humidity (40 ± 3%) were maintained throughout the fabrication process. The resultant PAN nanofiber membrane was dried under vacuum at 45 °C overnight, and then stored in a sealed container at room temperature for further use.

**Figure 2:**
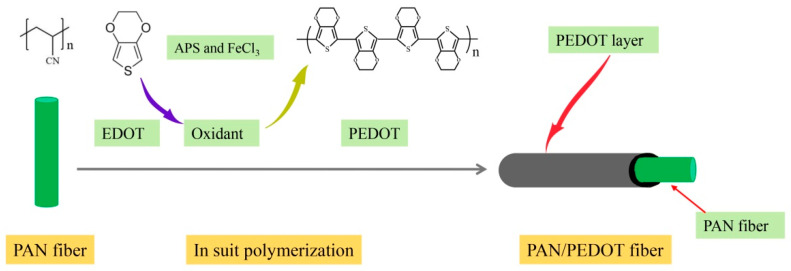
Fabrication of the PAN/PEDOT nanofiber with the in situ polymerization method.

PAN/PEDOT was fabricated by the in situ polymerization method. Figure 2 shows an illustration of the fabrication procedure for the preparation of PAN/PEDOT nanofiber. Briefly, 1 g PAN nanofiber membrane was completely immersed in 5 mL 20% ethanol solution, then 1 mL of EDOT was added. After gentle shaking, 10 mL APS and FeCl_3_ solution (molar ratio = 10/1) was added into the mixture, and the reaction mixture was kept at 30 °C overnight for oxidation. Finally, the PAN/PEDOT nanofiber was washed successively with excessive ethanol and ultrapure water under ultrasonic oscillation, and then dried in a vacuum oven at 40 °C overnight.

PS/PPY was also fabricated with the method similar to PAN/PEDOT. Briefly, 1 g PS nanofiber membrane was completely immersed in 5 mL 20% ethanol solution, then 1 mL pyrrole was added. After gentle shaking, 10 mL 0.1 M FeCl_3_ solution was added into the mixture, and the reaction mixture was kept at 30 °C overnight for oxidation. Finally, the PAN/PEDOT nanofiber was washed successively with excessive ethanol and ultrapure water under ultrasonic oscillation, and then dried in a vacuum oven at 40 °C overnight.

### 2.4. Preparation of Standard Stock Solution and Samples

Standard stock solutions were prepared for each acid at a concentration of 17.47 mM AA, 13.42 mM PA, 10.78 mM IBA, 10.94 mM BA, 9.07 mM IVA, 9.19 mM VA, 7.98 mM CA, 7.92 mM HXA, and 7.05 mM HPA, respectively. Then, 0.02 mmol/L 2-Ethylbutyric acid standard solution was also prepared by diluting 2-Ethylbutyric acid with water. All the standard stock solutions were stored at −20 °C until use. Working solutions were prepared by diluting the stock solution with water daily.

**Figure 3:**
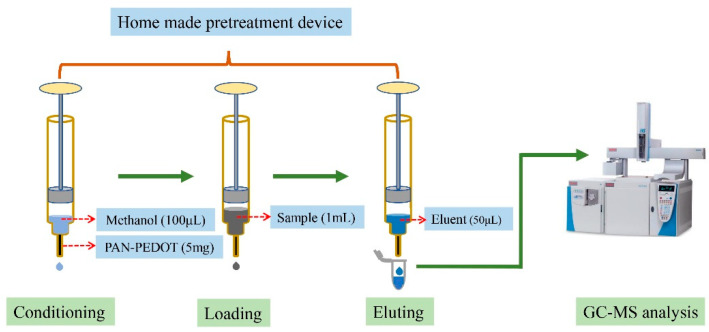
Flow chart of sample processing.

Sewage sludge collected from waste water treatment plants and fecal samples collected from healthy volunteers was used for developing and validating the present method. Ethical approval for this study was obtained from the Ethical Committee of Southeast University prior to the collection and analysis of these biological samples. The samples were frozen immediately after collection and stored at −20 °C until analysis. After thawing, 0.5 g sludge or fecal sample was suspended in 4.5 mL water, and shaken until a homogeneous suspension was formed. Then, the suspension was centrifuged at 12,000 rpm for 5 min, after which 1 mL supernatant and 10 μL 0.02 mmol/L internal standard were mixed and loaded to the SPE column (preconditioned with 100 μL methanol and water, respectively), which was packed with 5 mg PAN/PEDOT nanofibers. After the sample was pushed out of the column, the target compounds were then eluted with 50 μL of 0.01 mol/L hydrochloric acid ethanol solution (Figure 3). Finally, 1 μL eluent was injected into the GC–MS for analysis.

### 2.5. Experimental Conditions of GC–MS Analysis

Chromatographic analysis was carried out using Thermo Trace 1300 ISQ QD system (Madison, WI, USA). A fused-silica capillary column (30 m × 0.32 mm i.d.) coated with a 0.5 μm film thickness of polyethylene glycol phase (DB-WAX, J&W Scientific, Agilent Technologies Inc., Folsom, CA, USA) was used. Helium was supplied as the carrier gas at a flow rate of 1.5 mL/min. The initial oven temperature was 60 °C, maintained for 1 min, raised to 110 °C at 10 °C/min and held for 5 min, then increased to 161 °C at 3 °C/min, and finally held at 161 °C for 5 min. Injection was done in splitless mode with an injection volume of 1 μL and an injector temperature of 200 °C. It was operated in SIM mode, and with an ionization voltage of 70 eV. The ion source temperature was 300 °C and the interface temperature was 250 °C.

### 2.6. Method Validation and Application

SCFAs standard stock solution was diluted with water into a series of concentrations, and treated with the method mentioned above. Each concentration and the corresponding peak area were constructed for the calibration curves. Limits of detection (LODs) were calculated from the standard calibration curves by considering the peak area corresponding to three times the signal to noise ratio, while limits of quantification (LOQs) were 10 times the signal-to-noise ratio. The reproducibility of the method was evaluated by the intra-day and inter-day precisions at three concentration levels (0.1 μmol/L, 1 μmol/L, and 10 μmol/L), and the precision of the method was expressed in terms of relative standard deviation (RSD). For the recovery test, a known amount of SCFAs was added into the fecal samples, and samples were extracted and analyzed with the developed method. To verify the feasibility of this established method, waste water and fecal samples were collected from two waste water treatment plant and eight healthy volunteers, and then analyzed using the developed method.

## 3. Results

### 3.1. Characterization of the PAN/PEDOT Nanofiber

**Figure 4:**
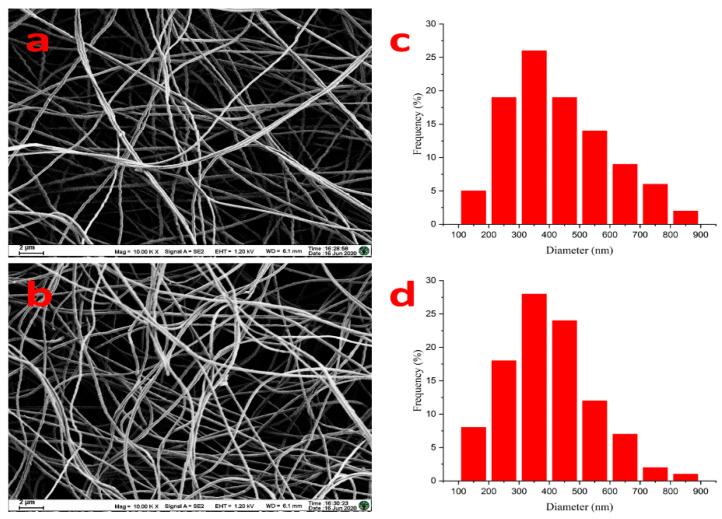
SEM images of electrospun nanofiber ((**a**) PAN; (**b**) PAN/PEDOT) and diameter distributions ((**c**) PAN; (**d**) PAN/PEDOT).

The morphology and structure of the prepared PAN/PEDOT nanofiber were observed by a Zeiss Ultra Plus scanning electron microscope (SEM) (Oberkochen, Germany). As shown in Figure 4, PAN nanofiber had a homogeneous and smooth morphology, and PAN/PEDOT composite fiber also had a network structure with high porosity. This indicated that the incorporation of PEDOT did not significantly change the fibrous morphology.

### 3.2. GC–MS Detection of SCFAs

**Figure 5:**
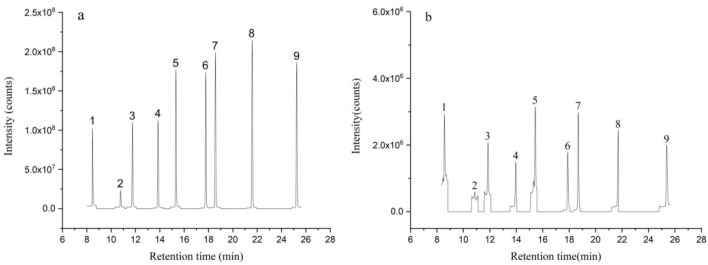
Total ion current chromatogram of a standard mixture of SCFAs (**a**) and eluent of the sample (**b**). (1) AA; (2) PA; (3) IBA; (4) BA; (5) IVA; (6) VA; (7) 2-ethylbutyric acid; (8) HXA; and (9) HPA.

SCFAs standard solution and sewage sludge solution were treated with the method mentioned above. The typical chromatograms of SCFAs standards and sample were obtained with GC–MS. Under optimal experimental conditions, SCFAs were isolated completely in 26 min (Figure 5). The retention time and parameters of quantification are shown in Table 1.

### 3.3. Validation of the Method

All calibration curves showed a good linearity (R^2^ ≥ 0.995) in a wide range of concentrations (Table 2). The limits of detection (LODs, S/N = 3) and the limits of quantification (LOQs, S/N = 10) are also shown in Table 2. In addition, the reproducibility of the method was evaluated by the intra-day and inter-day precisions at three concentration levels (0.1 μmol/L, 1 μmol/L, and 10 μmol/L). From Table 2, it can be found that the recoveries of SCFAs in fecal sample matrices ranged from 87.5% to 104.6%. The relative standard deviations of the method were 6.5–13.7% (*n* = 5). LODs and LOQs were 0.34–0.87 μmol/L and 1.14–2.87 μmol/L, respectively. The calibration curves of the analyte investigated in the range of 1.54–3331 μmol/L and the linear correlation coefficient (R^2^) between 0.9953 and 0.9991 were obtained.

### 3.4. Comparison with Other Methods

**Figure 6:**
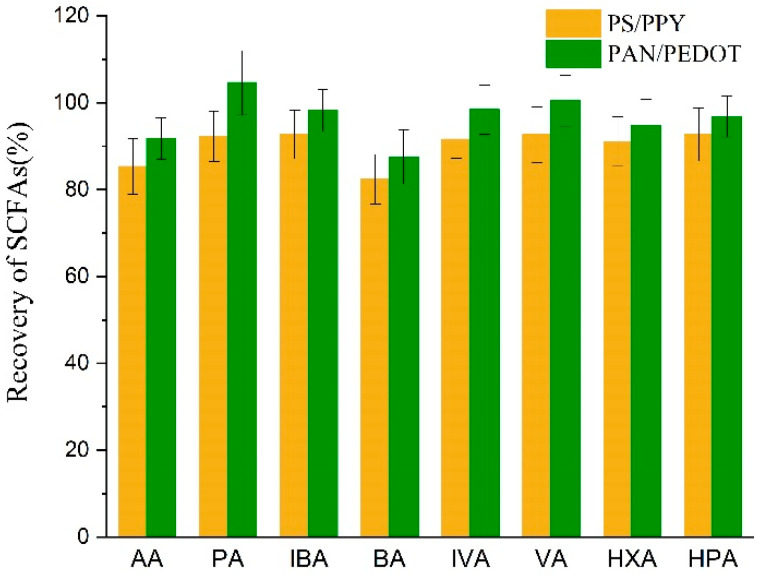
Recovery of SCFAs with PS/PPY and PAN/PEDOT.

A previous report from our group showed that polystyrene/polypyrrole (PS/PPY) can be successfully applied in the determination of SCFAs, with high selectivity and sensitivity [30]. However, this composite nanofiber membrane was hydrophobic, and is not an ideal absorbent for aqueous samples. In this work, a hydrophilic polymer PAN was selected as the substrate, and PEDOT as the coating. This composite nanofiber displayed higher recovery of SCFAs than PS/PPY (Figure 6).

Compared with other methods reported for SCFAs’ analysis (Table 3), which need long extraction time or lots of solvents, the proposed method only consumed 0.2 mL of organic solvent and was sensitive enough for quantification. Owing to the larger surface to volume ratio of the PAN nanofiber and the multiple action sites of conductive PEDOT coating, the composite nanofiber showed high extraction efficiency for SCFAs. The underlying mechanism for the adsorption of SCFAs on the fibers may be the interactions between the functionalized nanofiber and SCFAs, such as π–π interactions, hydrogen bonding, acid–base properties, and hydrophobic interactions [29,31,32]. More importantly, the whole pretreatment including sample extraction and enrichment can be finished in 3 min through the omitting of heating and evaporation steps for the concentration of the analytes, which could reduce the loss of volatile target compounds. The adsorbent materials can be prepared easily, which can meet the requirements of batch analysis of environmental samples.

### 3.5. Determination of SCFAs in Real Samples

Sewage sludge collected from waste water treatment plants and fecal samples collected from healthy volunteers were treated with PEDOT/PAN nanofiber and analyzed by GC–MS. The analytical results of waste water and fecal samples are given in Table 4.

## 4. Conclusions

In this work, a convenient, simple, and inexpensive method based on solid phase extraction of SCFAs with PAN/PEDOT nanofiber coupled with GC–MS was reported, and the proposed method was successfully applied for the determination of SCFAs in waste water and fecal samples. The advantages of the method are summarized below:

(1) A simple method to fabricate conductive nanofiber as the adsorbent for the extraction of SCFAs was established. Firstly, hydrophilic PAN nanofiber was produced by electrospinning, and then the PEDOT coating was deposited on the surface of the PAN nanofiber in aqueous solution through in situ polymerization. The PAN/PEDOT nanofibers had the potential to serve as a good sorbent material for SPE-based techniques because of their unique physical and chemical properties, such as large surface area to volume ratio and porousness, which were suitable for the attachment of target molecules. The nanofiber-based SPE only needed a little volume of solid phase, resulting in a small volume of eluting solvent, which could be injected into an analytical instrument without an evaporation step for concentration of the target compounds.

(2) The pretreatment of SCFAs in waste water and fecal samples was simple, fast, and efficient. The sample preparation procedure integrated separation, purification, and concentration into one step, which can minimize the loss of the analyte, and is suitable for volatile targets, such as SCFAs. The pretreatment process can be finished in 3 min for one sample, and used less organic solvent than conventional solid-phase extraction (SPE).

(3) Compared with the traditional sample handling procedure, which needs sulfuric acid [12] or phosphoric acid [35] to acidify and derive the sample before GC analysis, the new method using PAN/PEDOT nanofiber as the SPE sorbent can effectively isolate the SCFAs from the complex samples without consuming more organic solvents, and the targets can be concentrated, and thus can be determined directly without derivatization.

As a result, the new method had a wide linear range (from 1.54 μmol/L to 3331 μmol/L), excellent linearity (R^2^ > 0.9953), good recoveries (87.5–104.6%), and low LODs (0.34–0.87 µmol/L) and LOQs (1.14–2.87 µmol/L), which can meet the needs for trace analysis of SCFAs in complex samples. Owing to the feasibility and simplicity of the new method, it was concluded that this method has the potential for the detection of SCFAs in environmental and biological samples. In the future, we want to develop a more sensitive, simple, and reliable determination method of SCFAs to evaluate the health state of humans, as SCFAs have positive physiological effects on the human body.

## Figures and Tables

**Table 1:** Retention time and quantitative ion of SCFAs.

SCFAs	Retention Time	Quantitative Ion (*m*/*z*)
AA	8.41	43.1, 60.1
PA	10.73	57.1,73.1
IBA	11.71	41.1, 43.1
BA	13.85	42.1, 60.1
IVA	15.31	60.1, 87.1
VA	17.77	60.1, 73.1
HXA	21.58	73.1, 87.1
HPA	25.24	60.1

**Table 2:** Analytical performance of the method.

SCFAs	R^2^	Linear Range (μmol/L)	LOD (μmol/L)	LOQ (μmol/L)	Intra-Day RSD (%, *n* = 5)			Intra-Day RSD (%, *n* = 5)			Recovery ^b^ (%)
					0.1 ^a^	1 ^a^	10 ^a^	0.1 ^a^	1 ^a^	10 ^a^	
AA	0.9967	3.33–3331	0.87	2.87	9.7	8.6	7.9	10.2	9.7	7.1	91.7
PA	0.9980	2.70–1351	0.61	2.02	7.5	8.1	6.5	8.9	11.5	7.8	104.6
IBA	0.9969	2.27–1135	0.56	1.85	8.5	7.7	7.2	7.6	7.4	6.9	98.2
BA	0.9987	2.27–1135	0.49	1.62	6.9	10.1	9.7	6.9	13.1	13.4	87.5
IVA	0.9991	1.96–979	0.41	1.37	9.7	13.7	9.9	7.8	12.3	10.0	98.4
VA	0.9959	1.96–979	0.34	1.14	10.0	9.3	12.0	9.5	10.9	9.9	100.5
HXA	0.9974	1.72–861	0.38	1.26	7.4	8.7	9.2	7.8	8.9	7.2	94.7
HPA	0.9953	1.54–768	0.40	1.33	9.9	7.8	7.4	10.7	14.1	8.9	96.8

^a^ Unit of the spiked concentration of RSD: μmol/L. ^b^ Spiked concentration: 1 μmol/L.

**Table 3:** Comparison of different methods for SCFAs’ analysis.

Detection Method	Extraction or Derivatization	Pretreatment Time	Solvent Used	LOD (μmol/L)	LOQ (μmol/L)	Others	Ref.
GC–MS	Both were used	>100 min	0.4 mL	0.064–0.067	1.605–1.678	Dehydration was adopted before derivatizatiom	[33]
GC–FID	None was used	Not provided	Not provided	0.096–0.628	0.283–1.894	Two columns were used together	[34]
GC–FID	Solvent extraction	>18 min	3 mL	0.04–0.64	0.14–2.12	Extraction was repeated three times	[35]
GC–FID/MS	SPME	>20 min	0	0.068–11.24	0.62–105.58		[36]
GC–FID	None was used	>33 min	0	0.72–9.04	2.38–30.14		[37]
GC–MS	Ethanol/HCl extraction	<3 min	0.2 mL	0.34–0.87	1.14–2.87		This work

**Table 4:** SCFAs’ concentrations in real samples.

Sample	AA	PA	IBA	BA	IVA	VA	HXA	HPA
Waste water 1# (mg/L)	145.61	23.19	18.74	56.92	24.67	18.91	108.71	85.61
Waste water 2# (mg/L)	238.9	57.61	37.91	102.2	75.64	29.81	46.75	24.29
Waste water 3# (mg/L)	89.32	36.9	46.1	75.4	39.7	37.8	26.51	27.84
Waste water 4# (mg/L)	142.1	44.9	61.8	102.4	65.7	43.1	51.8	69.7
Waste water 5# (mg/L)	135.6	98.7	74.9	164.9	58.3	76.8	21.8	10.2
Waste water 6# (mg/L)	66.7	76.4	59.7	100.2	27.9	38.7	44.5	60.8
Waste water 7# (mg/L)	33.4	26.87	34.9	42.78	56.7	53.84	25.17	26.97
Waste water 8# (mg/L)	105.8	69.75	51.8	63	24.8	61.83	49.8	28.62
Fecal 1# (mmol/kg)	19.22	6.57	2.14	4.52	2.97	1.62	6.18	0.05
Fecal 2# (mmol/kg)	24.12	7.34	1.97	5.31	8.65	6.24	4.21	0.1
Fecal 3# (mmol/kg)	18.79	10.34	2.53	8.97	1.07	3.79	5.61	0.02
Fecal 4# (mmol/kg)	32.48	4.79	2.0	10.32	7.98	2.18	3.97	0.07
Fecal 5# (mmol/kg)	15.73	8.51	1.82	5.64	2.62	5.31	4.91	0.05
Fecal 6# (mmol/kg)	20.01	7.1	3.04	8.59	2.80	4.09	8.2	0.09

## Data Availability

The data presented in this study are available on request from the corresponding author.
